# A study of Mandelbrot and Julia Sets via Picard–Thakur iteration with *s*-convexity

**DOI:** 10.1371/journal.pone.0315271

**Published:** 2025-03-21

**Authors:** Bashir Nawaz, Krzysztof Gdawiec, Kifayat Ullah, Maggie Aphane

**Affiliations:** 1 Department of Mathematics, University of Lakki Marwat, Lakki Marwat, Khyber Pakhtunkhwa, Pakistan; 2 Institute of Computer Science, University of Silesia, Bedzinska, Sosnowiec, Poland; 3 Department of Mathematics and Applied Mathematics, Sefako Makgatho Health Sciences University, Pretoria, South Africa; Sefako Makgatho Health Sciences University Faculty of HealthSciences, SOUTH AFRICA

## Abstract

Nowadays, many researchers are employing various iterative techniques to analyse the dynamics of fractal patterns. In this paper, we explore the formation of Mandelbrot and Julia sets using the Picard–Thakur iteration process, extended with *s*-convexity. To achieve this, we establish an escape criterion using a complex polynomial of the form $x^{k+1}\;+\;c$, where *k* ≥ 1 and *x*, *c* ∈ *ℂ*. Based on our proposed algorithms, we provide graphical illustrations of the Mandelbrot and Julia sets. Additionally, we extend our research to examine the relationship between the sizes of Mandelbrot and Julia sets and the iteration parameters, utilising some well-known methods from the literature.

## 1 Introduction

Fractals play a significant role in representing features of our natural environment. Some naturally occurring fractal patterns include trees, clouds, mountains, fern leaves, snowflakes, lightning bolts, river networks, and many more. Moreover, fractal geometry is a popular branch of mathematical art, providing a framework for creating these complex objects. Fractals are not only visually fascinating but also have practical applications in various fields. In computer graphics, fractals are used to create realistic landscapes [1], textures [2], and artistic patterns [3]. Moreover, fractal analysis is employed in medical imaging [4], financial modelling [5], and even in the study of chaotic dynamical systems [6]. The study of fractals bridges the gap between abstract mathematics and intricate patterns observed in the natural world, providing a deeper understanding of both.

The study of fractals began in the early 20th century when P. Fatou and G. Julia sought successive approximations of the function $g(x)=x^{2}\;+\;c$, where *x*, *c* ∈ *ℂ*. In 1919, Julia [7] successfully iterated this function but could not visualise it. B. Mandelbrot [8] described the complex graphs of *g* and coined the term fractal. Mandelbrot sketched the Julia set and studied its characteristics, noting that the Julia sets exhibit diverse behaviours for different values of *c*. Mandelbrot introduced a new set called the Mandelbrot set by swapping the roles of *x* and *c* in the Julia set. The Mandelbrot set contains all values of *c* for which the corresponding Julia set is connected. This discovery was pivotal, as it divulged the intricate boundary of the Mandelbrot set, a hallmark of fractal geometry, showcasing self-similarity and infinite complexity. The properties of the Mandelbrot and Julia sets are widely studied in the literature. The most apparent generalisation [9,10] involves using the function $x^{p}+c$ instead of $x^{2}+c$. Other functions have also been examined in the literature, including transcendental functions [11], rational functions [12,13]. Additionally, the study of Mandelbrot sets has been extended from the complex number system to bicomplex numbers [14], quaternions [15], octonions [16], and more.

In the literature, Julia sets and Mandelbrot sets have also been generalised using various iteration processes derived from fixed point theory. Within fixed point theory, numerous approximation methods exist for approximating the fixed points of a given mapping, employing different feedback iteration processes. These methods can be applied to generalise Julia sets and Mandelbrot sets. The Mann and the Ishikawa iteration processes have been used by different authors [17–19] to outline the concepts of superior Julia and superior Mandelbrot sets. In [20], the authors utilised the Picard–Mann iteration process to examine the properties of Mandelbrot and Julia sets. In [21], the authors investigated the intricate properties of Mandelbrot and Julia sets by employing the M-iteration process. Similarly, in [22], the properties of these sets were explored using the Noor iteration process. The Picard–Thakur hybrid iteration was used to generate fractals for the polynomial function $z^{m}+b$ [23] and the transcendental functions [24]. Additionally, the literature includes examples of implicit iterations, such as the use of the Jungck–Mann iteration to generate Julia sets [25] and the Jungck–Noor implicit scheme to obtain images of the Mandelbrot and Julia sets [26].

Some researchers have extended the fixed point results using *s*-convexity to generate intricate structures of fractals. Convexity and its generalisations are crucial in various mathematical fields, particularly in optimisation theory. Some fixed point results with *s*-convexity include Ishikawa’s orbit with *s*-convexity [27], Jungck–Ishikawa’s orbit with *s*-convexity [28], Noor’s orbit with *s*-convexity [29], SP orbit with *s*-convexity [30], Picard–Mann with *s*-convexity and S iteration with *s*-convexity [31], Jungck–Noor orbit with *s*-convexity [32], and Jungck–CR iteration with *s*-convexity [33]. Additionally, Rawat et al. [34] generated Mandelbrot and Julia sets for generalised rational maps using SP iteration with *s*-convexity. Murali and Muthunagai [35] employed a viscosity approximation type iterative method extended with *s*-convexity to generate Mandelbrot and Julia sets for rational type mapping and Ahmad et al. [36] for the transcendental functions. Mishra et al. [27] explored the properties of tricorns and multicorns using Ishikawa iteration with *s*-convexity. Kwun et al. [37] used Noor iteration with *s*-convexity to generate tricorns and multicorn. Li et al. [38] employed CR iteration with *s*-convexity to produce antifractals.

Fascinated by the captivating images of fractals and intrigued to explore their generation using various iteration processes, the main motivation behind the presented contribution was to delve into the potential of utilising a new iteration scheme to create visually appealing fractal patterns and to analyse its behaviour.

By incorporating advanced mathematical concepts like *s*-convexity, in this paper, we adopt this approach to generate fascinating structures of Mandelbrot and Julia sets using the Picard–Thakur hybrid iteration process equipped with *s*-convexity. Through our proposed iteration process, we formulate a new escape criterion to generate Mandelbrot and Julia sets. We implement the proven criterion in the escape-time algorithms and present graphical examples obtained using these proposed algorithms. Moreover, we explore the relationship between the iteration parameters and the fractal properties to gain deeper insights and identify potential improvements in the field of complex dynamics and fractal geometry using some existing methods from the literature [39,40].

The remainder of the paper is organised as follows. In Sect 2, we present key definitions and fundamental facts necessary for the subsequent discussions. In Sect 3, we derive the escape criterion for the considered iteration scheme. Sect 4 presents graphical images of the Mandelbrot and Julia sets generated using the Picard–Thakur iteration equipped with *s*-convexity. In Sect 5, we investigate the relationships between iterative parameters and numerical measures. Finally, Sect 6 provides the study’s concluding remarks.

## 2 Preliminaries

In this section, we provide the key definitions essential to our main result.

**Definition 2.1** (Julia set [41]). Let ${\text{A}}_{c}:\mathbb{C}\to \mathbb{C}$ be a complex polynomial, where *c* ∈ *ℂ* is a parameter. The filled Julia set is defined as


$$J_{{\text{A}}_{c}}=\{x\in \mathbb{C}:{\left\{|{\text{A}}_{c}^{n}(x)|\right\}}_{n=0}^{\infty }\text{ is bounded}\},$$

where ${\text{A}}_{c}^{n}(x)$ represent the *n*th iterate of the polynomial ${\text{A}}_{c}$. The boundary of $J_{{\text{A}}_{c}}$ is called the Julia set.

**Definition 2.2** (Mandelbrot set [41]). Mandelbrot set includes all values of *c* for which the filled Julia set $J_{{\text{A}}_{c}}$ is connected, i.e.,


$$M=\{c\in \mathbb{C}:J_{{\text{A}}_{c}}\text{ is connected}\}.$$

Equivalently, the Mandelbrot set can be defined as [41]


$$M=\{c\in \mathbb{C}:|{\text{A}}_{c}^{n}(\omega )|↛\infty \text{ as }n\to \infty \},$$

where *ω* is a critical point of ${\text{A}}_{c}$, i.e, ${\text{A}}_{c}^{\prime }(\omega )=0$.

**Definition 2.3** (Picard–Thakur iteration [42]). Let G:X→X be a mapping. Consider a sequence $\left\{x_{n}\right\}$ of iterates for the initial point $x_{0}\in X$. We call the sequence $\left\{x_{n}\right\}$ the Picard–Thakur iteration process if


$$\begin{array}{l} \begin{array}{lll} v_{n} & =(1-\varrho_{n})x_{n}+\varrho_{n}Gx_{n} & \\ y_{n} & =(1-\psi_{n})v_{n}+\psi_{n}Gv_{n},\\ w_{n} & =(1-\sigma_{n})Gv_{n}+\sigma_{n}Gy_{n},\\ x_{n+1} & =Gw_{n}, \end{array} \end{array}$$ (1)

where $\sigma_{n},\psi_{n},\varrho_{n}\in (0,1]$ for all *n* ∈ *ℕ*.

**Definition 2.4** (*s*-convex combination [43]). Let $x_{1},x_{2},\ldots ,x_{n}\in \mathbb{C}$ and *s* ∈ (0, 1]. The *s*-convex combination is defined as:


$$\begin{array}{c} \lambda_{1}^{s}x_{1}+\lambda_{2}^{s}x_{2}+\ldots +\lambda_{n}^{s}x_{n}, \end{array}$$ (2)

where $\lambda_{i}\geq 0$ for *i* ∈ {1, 2, …, *n*} and $\sum_{i=1}^{n}\lambda_{i}=1$.

## 3 Escape criterion for the Picard–Thakur iteration with *s*-convexity

In the first three steps of the Picard–Thakur iteration defined in (1), we use the standard convex combination of two elements. We will extend this iteration by replacing the convex combination with the *s*-convex one. Moreover, we will employ it to the $\text{A}(x)=x^{k+1}\;+\;c$ function and use constant sequences of the parameters $\varrho_{n}$, $\psi_{n}$, $\sigma_{n}$. As a result, we get the following Picard–Thakur iteration scheme with *s*-convexity:


$$\begin{array}{c} \begin{array}{lll} v_{n} & ={\left(1-\varrho \right)}^{s}x_{n}+\varrho^{s}\text{A}(x_{n}), & \\ y_{n} & ={\left(1-\psi \right)}^{s}v_{n}+\psi^{s}\text{A}(v_{n}),\\ w_{n} & ={\left(1-\sigma \right)}^{s}\text{A}(v_{n})+\sigma^{s}\text{A}(y_{n}),\\ x_{n+1} & =\text{A}(w_{n}), \end{array} \end{array}$$ (3)

where *σ*, *ψ*, *ϱ* ∈ (0, 1].

Now, we can consider conditions under which the Picard–Thakur iteration extended with *s*-convexity escapes to infinity for a given starting point.

**Theorem 3.1.** *Let* $\text{A}(x)=x^{k+1}+c$, *where* *c* ∈ *ℂ* *and* *k* = 1, 2, 3, …. *Assume that* $x_{0}\in \mathbb{C}$ *and*


$$\begin{array}{ccc} |x_{0}|\geq |c|, & & \end{array}$$ (4)

$$\begin{array}{ccc} |x_{0}|>{\left(\frac{2}{s\varrho }\right)}^{\frac{1}{k}}, & & \end{array}$$ (5)

$$\begin{array}{ccc} |x_{0}|>{\left(\frac{2}{s\psi }\right)}^{\frac{1}{k}}, & & \end{array}$$ (6)

*where* *σ*, *ψ*, *ϱ* ∈ (0, 1]. *Then*, $|x_{n}|\to \infty$ *as* *n* → *∞*, *where* $\left\{x_{n}\right\}$ *is defined in* (3).

*Proof*. For *n* = 0, let us start by considering


$$|v_{0}|=|{\left(1-\varrho \right)}^{s}x_{0}+\varrho^{s}{\text{A}}_{c}(x_{0})|.$$

Being ${\text{A}}_{c}(x_{0})=x_{0}^{k+1}+c$, we have


$$\begin{array}{llll} \left|v_{0}\right| & =|{\left(1-\varrho \right)}^{s}x_{0}+\varrho^{s}(x_{0}^{k+1}+c)|\; & & \;\\ & \geq |{\left(1-\varrho \right)}^{s}x_{0}+s\varrho (x_{0}^{k+1}+c)|∵\varrho^{s}\geq s\varrho \text{ as }s,\varrho \in (0,1]\; & & \;\\ & \geq |s\varrho (x_{0}^{k+1}+c)|-|{\left(1-\varrho \right)}^{s}x_{0}|.\; & & \; \end{array}$$

Because ${\left(1-\varrho \right)}^{s}\leq 1-s\varrho$ for *s*, *ϱ* ∈ (0, 1], we get


$$\begin{array}{lll} \left|v_{0}\right| & \geq |s\varrho (x_{0}^{k+1}+c)|-|(1-s\varrho )x_{0}| & \\ & \geq |s\varrho x_{0}^{k+1}|-|(1-s\varrho )x_{0}|-|s\varrho c|\\ & \geq |s\varrho x_{0}^{k+1}|-|(1-s\varrho )x_{0}|-|s\varrho x_{0}|\;\;∵|x_{0}|\geq |c|\\ & =s\varrho |x_{0}^{k+1}|-|x_{0}|+s\varrho |x_{0}|-s\varrho |x_{0}|\\ & =|x_{0}|(s\varrho |x_{0}{|}^{k}-1). \end{array}$$

Since $|x_{0}|>{\left(\frac{2}{s\varrho }\right)}^{\frac{1}{k}}$, we obtain


$$\begin{array}{ccc} s\varrho |x_{0}{|}^{k} & >2 & \\ s\varrho |x_{0}{|}^{k}-1 & >1\\ \left|x_{0}|(s\varrho |x_{0}{|}^{k}-1\right) & >|x_{0}|. \end{array}$$

Thus,


$$|v_{0}|\geq |x_{0}|.$$

Now, proceeding to the second step of the iteration process defined in (3), we get


$$\begin{array}{lll} \left|y_{0}\right| & =|{\left(1-\psi \right)}^{s}v_{0}+\psi^{s}\text{A}(v_{0})| & \\ & =|{\left(1-\psi \right)}^{s}v_{0}+\psi^{s}(v_{0}^{k+1}+c)|\\ & \geq |{\left(1-\psi \right)}^{s}v_{0}+s\psi (v_{0}^{k+1}+c)|∵\psi^{s}\geq s\psi \\ & \geq |s\psi (v_{0}^{k+1}+c)|-|{\left(1-\psi \right)}^{s}v_{0}|. \end{array}$$

Because ${\left(1-\psi \right)}^{s}\leq 1-s\psi$ for *s*, *ψ* ∈ (0, 1], we get


$$\begin{array}{lll} \left|y_{0}\right| & \geq |s\psi (v_{0}^{k+1}+c)|-|(1-s\psi )v_{0}| & \\ & \geq |s\psi v_{0}^{k+1}|-|s\psi c|-|(1-s\psi )v_{0}|\\ & \geq |s\psi v_{0}^{k+1}|-|s\psi v_{0}|-|(1-s\psi )v_{0}|\;\;∵|v_{0}|\geq |x_{0}|\geq |c|\\ & =|v_{0}|(s\psi |v_{0}{|}^{k}-1). \end{array}$$

Since $|v_{0}|>|x_{0}|>{\left(\frac{2}{s\psi }\right)}^{\frac{1}{k}}$, we obtain


$$\begin{array}{ccc} s\psi |v_{0}{|}^{k} & >2 & \\ s\psi |v_{0}{|}^{k}-1 & >1\\ \left|v_{0}|(s\psi |v_{0}{|}^{k}-1\right) & >|v_{0}|. \end{array}$$

Thus,


$$\begin{array}{c} |y_{0}|\geq |v_{0}|\geq |x_{0}|. \end{array}$$ (7)

Now, for


$$\begin{array}{lll} \left|w_{0}\right| & =|{\left(1-\sigma \right)}^{s}\text{A}(v_{0})+\sigma^{s}\text{A}(y_{0})| & \\ & =|{\left(1-\sigma \right)}^{s}(v_{0}^{k+1}+c)+\sigma^{s}(y_{0}^{k+1}+c)|\\ & \geq |{\left(1-\sigma \right)}^{s}(v_{0}^{k+1}+c)+s\sigma (y_{0}^{k+1}+c)|∵\sigma^{s}\geq s\sigma \text{ as }s,\sigma \in (0,1]\\ & \geq |s\sigma (y_{0}^{k+1}+c)|-|{\left(1-\sigma \right)}^{s}(v_{0}^{k+1}+c)|. \end{array}$$

Because ${\left(1-\psi \right)}^{s}\leq 1-s\psi$ for *s*, *ψ* ∈ (0, 1] and (7), we get


$$\begin{array}{lll} \left|w_{0}\right| & \geq |s\sigma (v_{0}^{k+1}+c)|-|(1-s\sigma )(v_{0}^{k+1}+c)| & \\ & =|s\sigma (v_{0}^{k+1}+c)|-|(s\sigma -1)(v_{0}^{k+1}+c)|\\ & \geq |s\sigma v_{0}^{k+1}|-|s\sigma c|-s\sigma |v_{0}^{k+1}|-s\sigma |c|+|v_{0}^{k+1}+c|\\ & =|v_{0}^{k+1}+c|\\ & \geq |v_{0}{|}^{k+1}-|c|\\ & \geq |x_{0}{|}^{k+1}-|x_{0}|\;\;∵|v_{0}|\geq |x_{0}|\geq |c|\\ & =|x_{0}|(|x_{0}{|}^{k}-1). \end{array}$$

Since $|x_{0}|>{\left(\frac{2}{s\varrho }\right)}^{\frac{1}{k}}>2^{\frac{1}{k}}$, so we get


$$\begin{array}{ccc} |x_{0}{|}^{k}-1 & >1 & \\ \left|x_{0}|(|x_{0}{|}^{k}-1\right) & >|x_{0}|. \end{array}$$

Thus,


$$|w_{0}|\geq |x_{0}|.$$

Now, for the fourth step of the iteration process (3), we have


$$\begin{array}{lll} \left|x_{1}\right| & =|\text{A}(w_{0})|=|w_{0}^{k+1}+c| & \\ & \geq |w_{0}^{k+1}|-|c|\\ & \geq |x_{0}^{k+1}|-|x_{0}|∵|w_{0}|\geq |x_{0}|\geq |c|\\ & =|x_{0}|(|x_{0}{|}^{k}-1). \end{array}$$

Since $|x_{0}|>{\left(\frac{2}{s\varrho }\right)}^{\frac{1}{k}}>2^{\frac{1}{k}}$, so we get


$$|x_{0}{|}^{k}-1>1.$$

So there exists *η* > 0 such that $|x_{0}{|}^{k}-1>1+\eta >1$. As a result, we obtain


$$|x_{1}|>|x_{0}|(1+\eta ).$$

In particular $\left|x_{1}|\geq |x_{0}\right|$, thus we may apply the same arguments repeatedly to obtain


$$\begin{array}{lll} \left|x_{2}\right| & >|x_{0}|{\left(1+\eta \right)}^{2}, & \\ \left|x_{3}\right| & >|x_{0}|{\left(1+\eta \right)}^{3},\\ & \vdots \\ \left|x_{n}\right| & >|x_{0}|{\left(1+\eta \right)}^{n}. \end{array}$$

Hence $|x_{n}|\to \infty$ as *n* → *∞*. □

From Theorem 3.1, we obtain the following corollaries.

**Corollary 3.2.** *Let* $\text{A}(x)=x^{k+1}+c$, *k* = 1, 2, 3, … *and* *c* ∈ *ℂ*. *Suppose that*


$$\begin{array}{c} |x_{0}|>\max\left\{|c|,{\left(\frac{2}{s\varrho }\right)}^{\frac{1}{k}}{\left(\frac{2}{s\psi }\right)}^{\frac{1}{k}}\right\}. \end{array}$$ (8)

*Then, for* $\left\{x_{n}\right\}$*defined by* (3), *we have* $|x_{n}|\to \infty$ *as* *n* → *∞*.

**Corollary 3.3.** *Let* $\text{A}(x)=x^{k+1}\;+\;c$, *where* *c* ∈ *ℂ* *and* *k* = 1, 2, 3, …. *Suppose that for* $\left\{x_{n}\right\}$ *defined in* (3), *we have*


$$\begin{array}{c} |x_{j}|>\max\left\{|c|,{\left(\frac{2}{s\varrho }\right)}^{\frac{1}{k}}{\left(\frac{2}{s\psi }\right)}^{\frac{1}{k}}\right\}\text{ for some }j\geq 0. \end{array}$$ (9)

*Then, there exists* *η* > 0 *such that* $|x_{n+j}|>|x_{j}|{\left(1+\eta \right)}^{n}$, *and we have* $|x_{n}|\to \infty$ *as* *n* → *∞*.

Corollary 3.3 is the so-called escape criterion, and it is the base for the escape-time algorithms used to generate Mandelbrot and Julia sets of the function $\text{A}(x)=x^{k+1}\;+\;c$ via the Picard–Thakur iteration with *s*-convexity, which we present in the next section.

## 4 Graphical examples

In this section, based on the escape criterion proved in Sect 3, we introduce the escape-time algorithms for generating Mandelbrot and Julia sets using the proposed Picard–Thakur iteration scheme with *s*-convexity. Additionally, we provide examples of sets generated by the algorithms.

### 4.1 Examples of Mandelbrot sets

Using Corollary 3.3, we can introduce an escape-time algorithm for generating a Mandelbrot set. The algorithm uses the escape criterion to determine whether the point calculated during each iteration escapes to infinity. The pseudocode of the algorithms is presented in Algorithm 1.

**Algorithm 1**: **Mandelbrot set.**



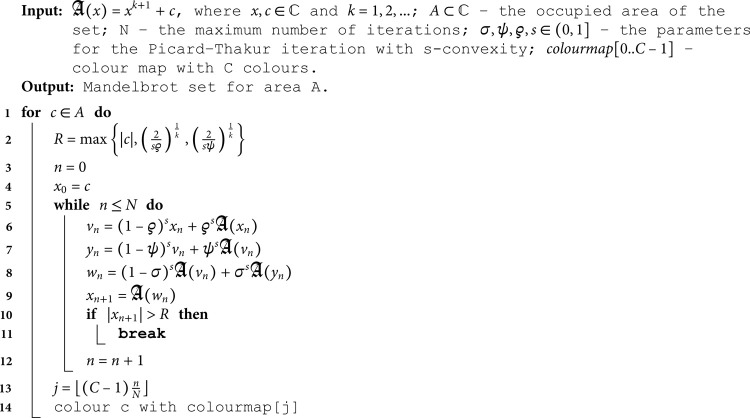



### 4.1.1 Quadratic Mandelbrot sets (*k* = 1)

**Fig 1:**
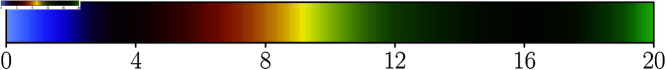
Colour map used in the graphical examples.

For the quadratic Mandelbrot set examples, we used *N* = 20 and a colour map given in Fig 1.

**Fig 2:**
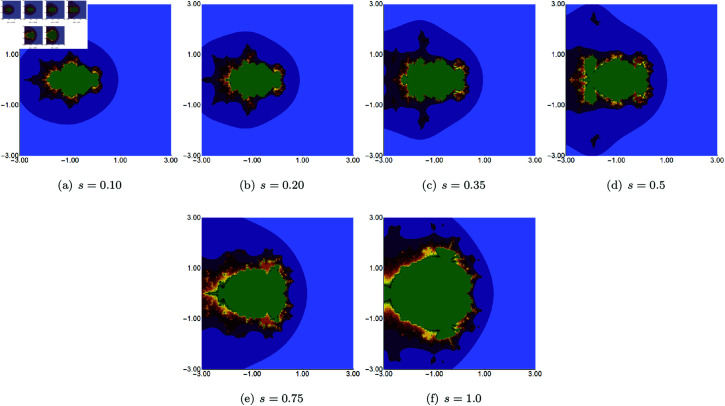
Quadratic Mandelbrot set generated using the Picard–Thakur iteration with *s*-convexity with *σ* = *ψ* = *ϱ* = 0.4 and varying values of *s.*

We start with quadratic Mandelbrot sets generated in $A={\left[-3,3\right]}^{2}$ using fixed values of *σ*, *ψ*, *ϱ*, and varying values of *s*. The parameters *σ*, *ψ*, and *ϱ* were fixed at 0.4. The images obtained are presented in Fig 2. From the images, we see that the *s* parameter significantly impacts the size of the sets. For high values of *s*, the set size is larger than for the lower values. The shape of the sets changes as the value of *s* varies. For *s* = 1.0, we see a large main bulb with a width-to-height ratio close to 1.0. As *s* becomes smaller, the main bulb is elongated, and a significant change in shape is observed, especially in the tail of the bulb. For all values of *s*, the sets exhibit axial symmetry, with the real axis serving as the symmetry line.

**Fig 3:**
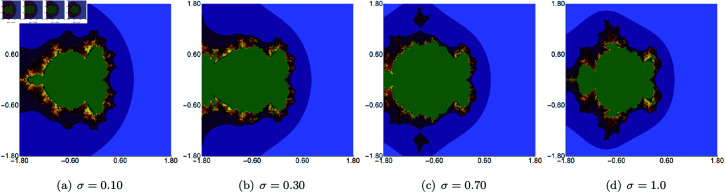
Quadratic Mandelbrot set generated using the Picard–Thakur iteration with *s*-convexity with *ψ* = *ϱ* = 0.6, *s* = 0.5 and varying values of *σ.*

**Fig 4:**
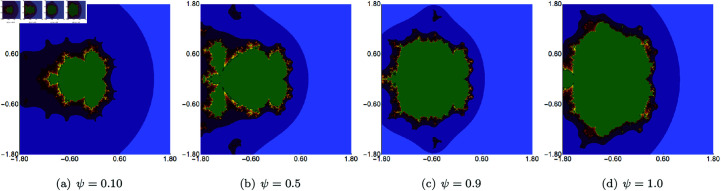
Quadratic Mandelbrot set generated using the Picard–Thakur iteration with *s*-convexity with *σ* = *ϱ* = 0.4, *s* = 0.5 and varying values of *ψ.*

**Fig 5:**
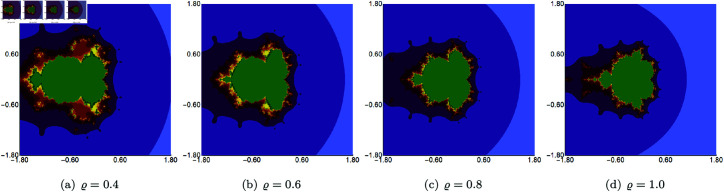
Quadratic Mandelbrot set generated using the Picard–Thakur iteration with *s*-convexity with *σ* = *ψ* = 0.2, *s* = 0.5 and varying values of *ϱ.*

In the next example, we fix the value of *s* to 0.5 and vary the other three parameters of the Picard–Thakur iteration with *s*-convexity. In Figs 3, 4, and 5, we present Mandelbrot sets obtained by varying *σ*, *ψ* and *ϱ*, respectively. The area for all sets was set to $A={\left[-1.8,1.8\right]}^{2}$. In each case, we can observe that the varying parameter impacts the size and shape of the generated set, but the changes are different. The smallest changes are visible for varying *ϱ* parameter, where we observe a gradual change in both the size and shape of the set. On the other hand, the largest changes are observed for varying values of *ψ*. For high values of *ψ*, we see a large set, and when we decrease the parameter’s value, the set’s size decreases. As in the case of varying *s*, we see that the sets have axial symmetry with the real axis as the symmetry axis.

### 4.1.2 Cubic Mandelbrot sets (*k* = 2)

In illustrating the cubic Mandelbrot sets, we set *N* = 20, maintaining the same colour map used for the quadratic case (Fig 1). The examples are organised in a manner analogous to the quadratic Mandelbrot sets.

**Fig 6:**
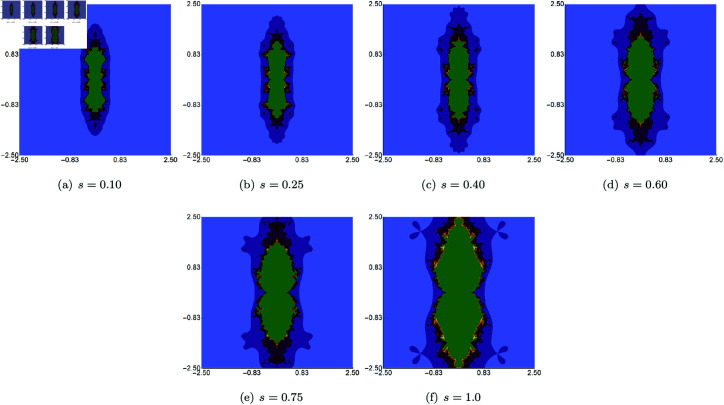
Cubic Mandelbrot set generated using the Picard–Thakur iteration with *s*-convexity with *σ* = *ψ* = *ϱ* = 0.5 and varying values of *s.*

We begin with examples where the parameters *σ*, *ψ*, and *ϱ* are fixed at 0.5, while the *s* parameter varies in the Picard–Thakur iteration with *s*-convexity. The area designated for the generation algorithm is $A={\left[-2.5,2.5\right]}^{2}$. The results produced by Algorithm 1 are shown in Fig 6. From these images, we observe that in the cubic Mandelbrot set, the *s* parameter exerts the greatest influence on the set’s size. The smallest set is obtained for low values of *s*, and when the parameter’s value increases, the set grows in size, obtaining the largest size for *s* = 1.0. When we look at the shapes of the sets, then we notice that the changes are minor, and the overall shape is very similar for all values of *s*. Additionally, we can see that for all values of *s*, the sets exhibit a 2-fold symmetry.

**Fig 7:**
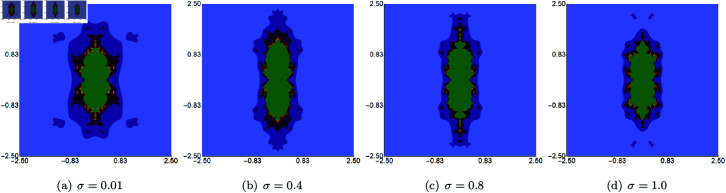
Cubic Mandelbrot set generated using the Picard–Thakur iteration with *s*-convexity with *ψ* = *ϱ* = 0.6, *s* = 0.5 and varying values of *σ.*

**Fig 8:**
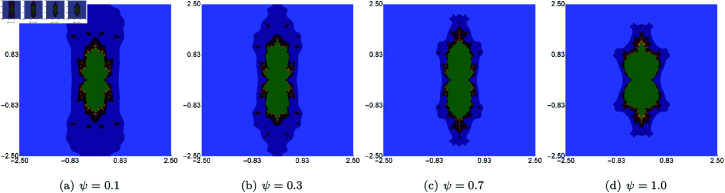
Cubic Mandelbrot set generated using the Picard–Thakur iteration with *s*-convexity with *σ* = *ϱ* = 0.6, *s* = 0.5 and varying values of *ψ.*

**Fig 9:**
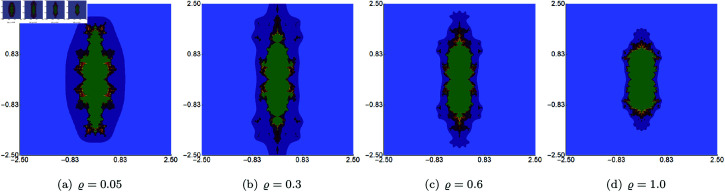
Cubic Mandelbrot set generated using the Picard–Thakur iteration with *s*-convexity with *σ* = *ψ* = 0.6, *s* = 0.5 and varying values of *ϱ.*

In the second set of examples, we held *s* constant at 0.5, along with two of the three parameters (*σ*, *ψ*, *ϱ*), allowing the third parameter to vary. The sets were generated within the area $A={\left[-2.5,2.5\right]}^{2}$. The outcomes of varying *σ*, *ψ*, *ϱ* are depicted in Figs 7, 8, and 9, respectively. These images reveal that alterations in the sets’ size are less substantial than those observed with changes to the *s* parameter. The most noticeable size change is observed for the varying *ϱ*, where we observe that the set is becoming more and more elongated as we decrease the value of the parameter. However, the boundaries of the sets exhibit distinct transformations with each parameter adjustment. For each of the varying parameters, we observe that the obtained sets have a 2-fold symmetry.

### 4.1.3 Quartic Mandelbrot sets (*k* = 3)

For all examples of quartic Mandelbrot sets, we used *N* = 20 and the same colour map as for the quadratic and cubic case (Fig 1). We divide the examples in a similar fashion as in the quadratic Mandelbrot sets.

**Fig 10:**
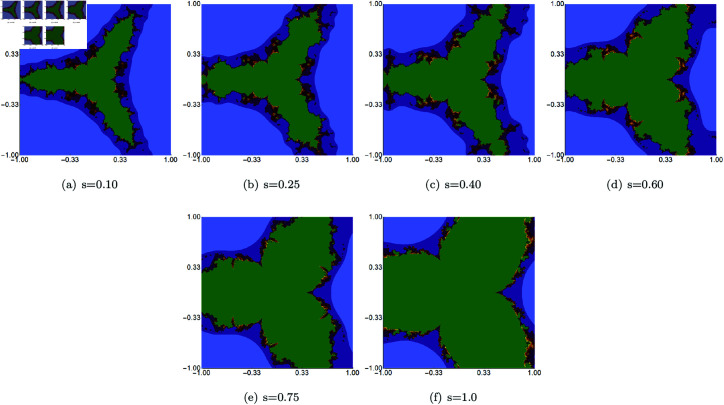
Quartic Mandelbrot set generated using the Picard–Thakur iteration with *s*-convexity with *σ* = *ψ* = *ϱ* = 0.5 and varying values of *s.*

We start with examples in which we fix the values of the *σ*, *ψ*, and *ϱ* parameters and vary the *s* parameter in the Picard–Thakur iteration with *s*-convexity. All fixed parameters were set to the same value of 0.5. The area for the generation algorithm was set to $A={\left[-1.0,1.0\right]}^{2}$. The results obtained with Algorithm 1 are presented in Fig 10. The images show that in the quartic Mandelbrot set, the *s* parameter significantly impacts the size of the set. The lower the value of *s*, the smaller the set size. The shape of the set does not change in a significant way. The changes are gradual. Moreover, we can observe that the sets have a 3-fold symmetry.

**Fig 11:**
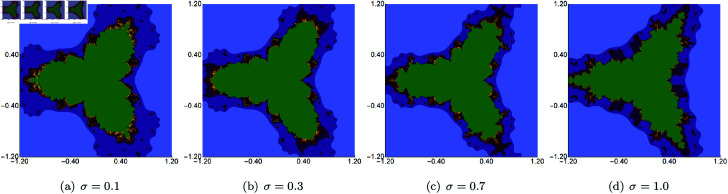
Quartic Mandelbrot set generated using the Picard–Thakur iteration with *s*-convexity with *ψ* = *ϱ* = 0.5, *s* = 0.5 and varying values of *σ.*

**Fig 12:**
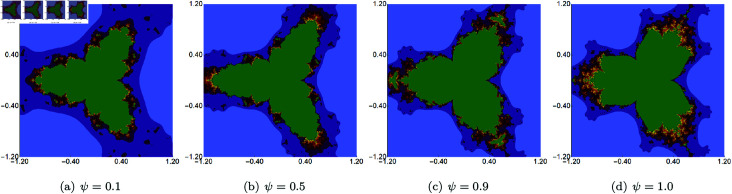
Quartic Mandelbrot set generated using the Picard–Thakur iteration with *s*-convexity with *σ* = 0.4, *ϱ* = 0.7, *s* = 0.5 and varying values of *ψ.*

**Fig 13:**
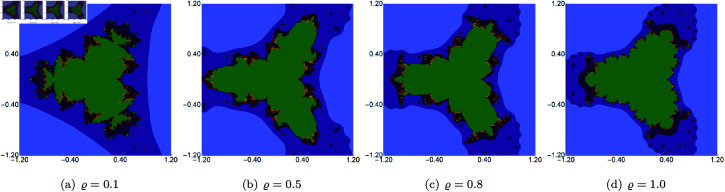
Quartic Mandelbrot set generated using the Picard–Thakur iteration with *s*-convexity with *σ* = 0.6, *ψ* = 0.2, *s* = 0.5 and varying values of *ϱ.*

In the second group of examples, we fixed the value of *s* and two of the three parameters (*σ*, *ψ*, *ϱ*) and varied the remaining parameter. This time, we generated the sets in the $A={\left[-1.2,1.2\right]}^{2}$ area, and the fixed value of *s* was set to 0.5. The results of changing the values of *σ*, *ψ*, *ϱ* are presented in Figs 11, 12, and 13, respectively. From the images, we can observe that the change in the size of the sets is not as big as in the case of varying the *s* parameter. We also see that the boundaries of the sets change gradually for each of the three parameters. The largest changes are visible for varying *ϱ*. Moreover, we see that each set has a 3-fold symmetry.

## 4.2 Examples of Julia sets

The escape criterion proved in Corollary 3.3 can be used to introduce an escape-time algorithm for the Julia sets via Picard–Thakur iteration with *s*-convexity. The pseudocode of this algorithm is presented in Algorithm 2.

**Algorithm 2**: **Julia set.**



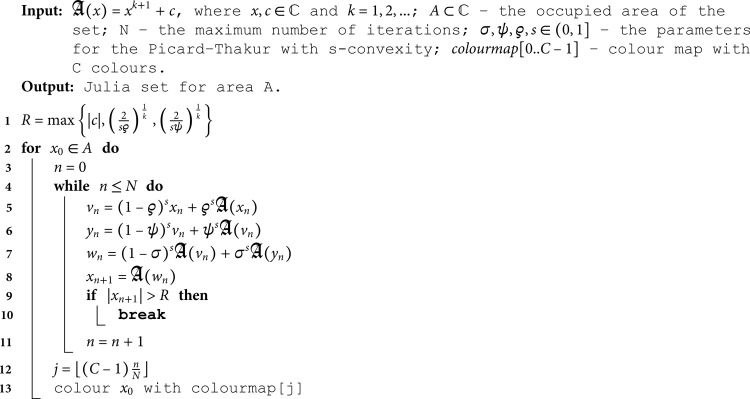



### 4.2.1 Quadratic Julia sets (*k* = 1)

To generate examples for the quadratic Julia sets, we used the following common parameters: *c* = − 0.57i, *N* = 20, $A={\left[-2,2\right]}^{2}$, and the colour map presented in Fig 1.

**Fig 14:**
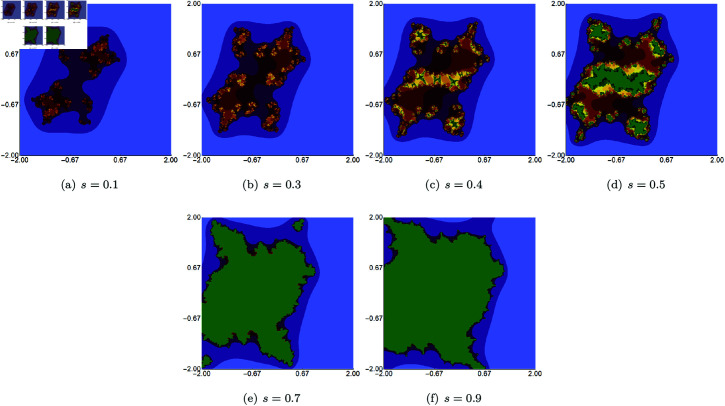
Quadratic Julia set for *c* = − 0.57i generated using the Picard–Thakur iteration with *s*-convexity with *σ* = *ψ* = *ϱ* = 0.4 and varying values of *s.*

We start the examples with quadratic Julia sets generated using Picard–Thakur iteration with *s*-convexity using fixed *σ*, *ψ*, *ϱ* parameters and varying the parameter responsible for the *s*-convexity, i.e., the *s* parameter. The fixed parameters were all set to 0.4. The images obtained are presented in Fig 14. When we look at the images, we observe that the *s* parameter impacts not only shape and size but also the connectivity of the set. For high values of *s*, the sets are larger, have simpler shapes, and are connected. The lower the value of *s*, the smaller the set becomes, and starting from 0.5, we see that the set loses its connectivity.

**Fig 15:**
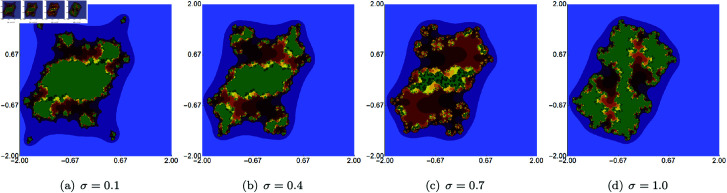
Quadratic Julia set for *c* = − 0.57i generated using the Picard–Thakur iteration with *s*-convexity with *ψ* = *ϱ* = 0.5, *s* = 0.5 and varying values of *σ.*

**Fig 16:**
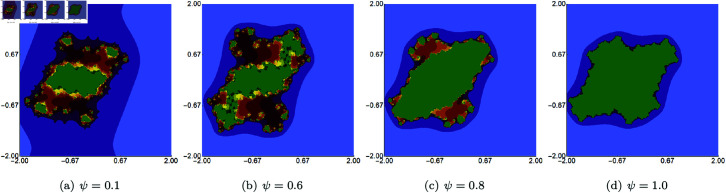
Quadratic Julia set for *c* = − 0.57i generated using the Picard–Thakur iteration with *s*-convexity with *σ* = *ϱ* = 0.5, *s* = 0.5 and varying values of *ψ.*

**Fig 17:**
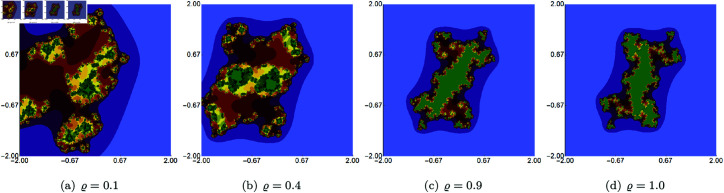
Quadratic Julia set for *c* = − 0.57i generated using the Picard–Thakur iteration with *s*-convexity with *σ* = *ψ* = 0.5, *s* = 0.5 and varying values of *ϱ.*

In the second example, we fixed the values of *s* and two of the three iteration parameters (*σ*, *ψ*, *ϱ*) and varied the remaining parameter. The value of *s* was to 0.5. The generated images for varying *σ*, *ψ*, *ϱ* are presented in Figs 15, 16, and 17, respectively. For each of the varying parameters, we observe a different change in size and shape. For the varying *σ*, we see the lower the value of the parameter, the more green points in the image, which shows that the filled Julia set is larger. For the other two cases, we see the opposite situation, i.e., the higher the value of *s*, the larger the filled Julia set. The complexity of the sets’ shapes has a reversed behaviour. For values for which the filled Julia sets are larger, we get simpler shapes of the sets.

### 4.2.2 Cubic Julia sets (*k* = 2)

**Fig 18:**
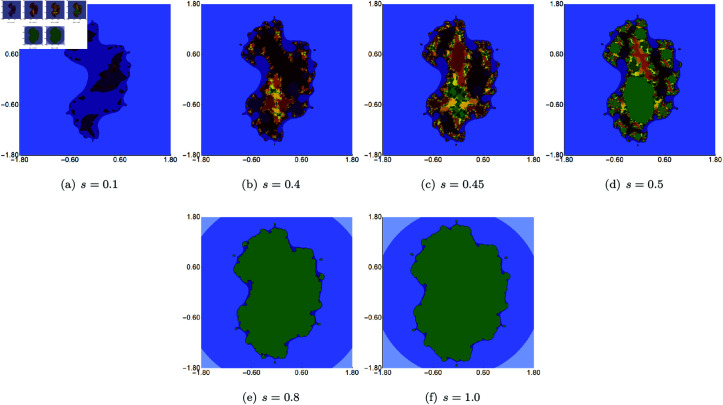
Cubic Julia set for *c* = − 0.3890 − 0.1859i generated using the Picard–Thakur iteration with *s*-convexity with *σ* = *ψ* = *ϱ* = 0.5 and varying values of *s.*

**Fig 19:**
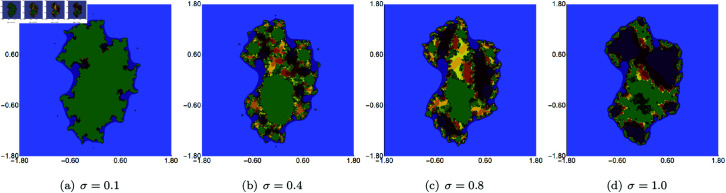
Cubic Julia set for *c* = − 0.3890 − 0.1859i generated using the Picard–Thakur iteration with *s*-convexity with *ψ* = *ϱ* = 0.5, *s* = 0.5 and varying values of *σ.*

**Fig 20:**
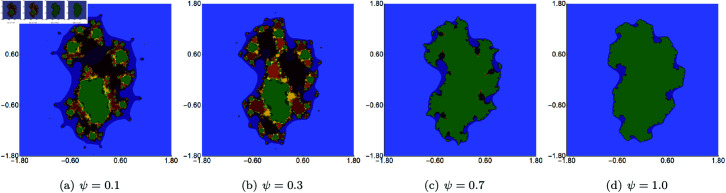
Cubic Julia set for *c* = − 0.3890 − 0.1859i generated using the Picard–Thakur iteration with *s*-convexity with *σ* = *ϱ* = 0.5, *s* = 0.5 and varying values of *ψ.*

**Fig 21:**
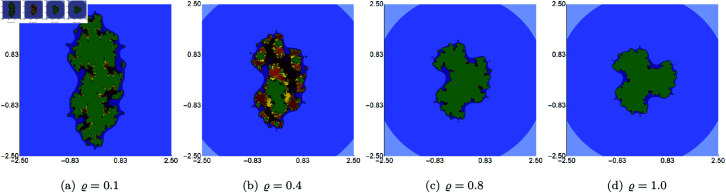
Cubic Julia set for *c* = − 0.3890 − 0.1859i generated using the Picard–Thakur iteration with *s*-convexity with *σ* = *ψ* = 0.5, *s* = 0.5 and varying values of *ϱ.*

For generating examples of cubic Julia sets, we employed the following common parameters: *c* = − 0.3890 − 0.1859i, *N* = 20 and the colour map shown in Fig 1. For Figs 18, 19, and 20, we used area $A={\left[-1.8,1.8\right]}^{2}$, whereas for Fig 21, we used $A={\left[-2.5,2.5\right]}^{2}$.

We begin by examining cubic Julia sets produced via Picard–Thakur iteration with *s*-convexity, using fixed values for *σ*, *ψ*, *ϱ*, while varying the parameter that controls *s*-convexity, specifically the *s* parameter. The fixed parameters were all set to 0.5. The resulting images are shown in Fig 18. From the images, we see that the *s* parameter affects not only the shape and size but also the set’s connectivity. For higher *s* values, the sets are larger, with simpler shapes and are connected. As *s* decreases, the sets shrink. This progression highlights the sensitivity of Julia sets to variations in the convexity parameter *s*. Moreover, none of the sets exhibits any type of symmetry.

In the second example, we fixed *s* at 0.5 and held two of the three iteration parameters (*σ*, *ψ*, *ϱ*) constant, while selectively varying the third. The resulting images, illustrating variations in *σ*, *ψ*, *ϱ*, are displayed in Figs 19, 20, and 21, respectively. Each parameter imparts a distinct influence on both the size and form of the set. For *σ*, lower values introduce more green points, signifying an expansion of the filled Julia set. Conversely, variations in *ψ* and *ϱ* demonstrate an opposite trend, where higher parameter values yield a larger filled Julia set. The intricacy of the shapes also follows a contrasting pattern: as the filled Julia sets grow, their forms become progressively simpler. This dynamic interplay between parameter values and structural complexity offers insights into the nuanced impact of iterative processes on fractal geometry.

### 4.2.3 Quartic Julia sets (*k* = 3)

For the examples generated for the quartic Julia sets, we used the following common parameters: *c* = − 1.0 + 0.02i, *N* = 20, $A={\left[-1.2,1.2\right]}^{2}$, and the colour map presented in Fig 1.

**Fig 22:**
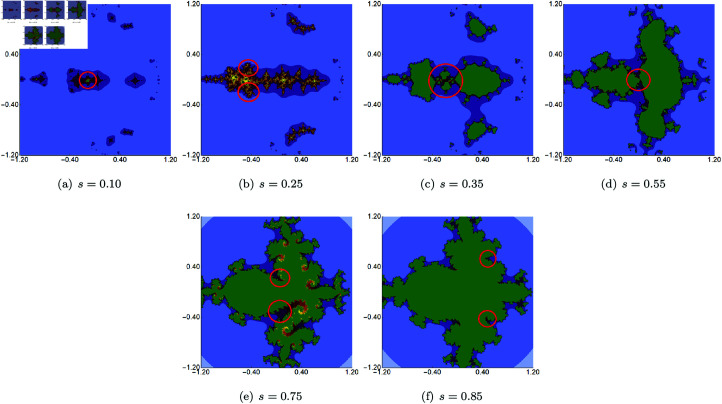
Quartic Julia set for *c* = − 1.0 + 0.02i generated using the Picard–Thakur iteration with *s*-convexity with *σ* = 0.7, *ψ* = 0.7, *ϱ* = 0.7 and varying values of *s.*

As in the previous examples, we start with an example where we vary the *s* parameter and fix the *σ*, *ψ*, and *ϱ* parameters. We set the fixed parameters to the following values: *σ* = 0.7, *ψ* = 0.7 and *ϱ* = 0.7. The generated images of the sets are presented in Fig 22. When we look at the images, we see a significant change in the size and shape of the sets with the value change of *s*. For high values of *s*, the set is larger and becomes smaller with the decrease of *s*. At first sight, it might seem that the sets have axial symmetry, but when we look closer, then, we notice areas that break the symmetry (see the exemplary areas marked in red).

**Fig 23:**
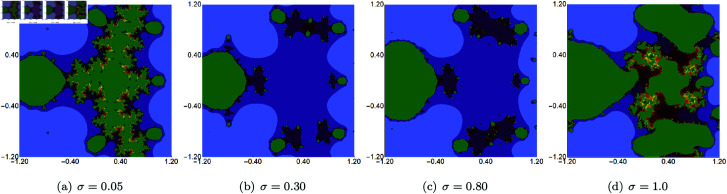
Quartic Julia set for *c* = − 1.0 + 0.02i generated using the Picard–Thakur iteration with *s*-convexity with *ψ* = *ϱ* = 0.10, *s* = 0.7 and varying values of *σ.*

**Fig 24:**
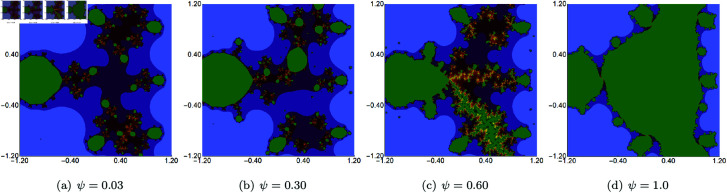
Quartic Julia set for *c* = − 1.0 + 0.02i generated using the Picard–Thakur iteration with *s*-convexity with *σ* = *ϱ* = 0.10, *s* = 0.7 and varying values of *ψ.*

**Fig 25:**
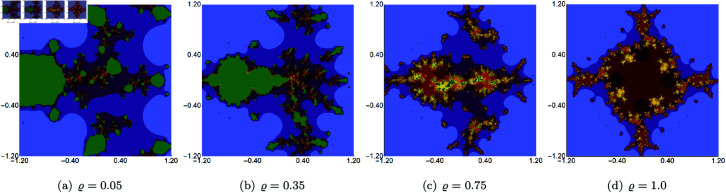
Quartic Julia set for *c* = − 1.0 + 0.02i generated using the Picard–Thakur iteration with *s*-convexity with *σ* = 0.10, *ψ* = 0.40, *s* = 0.7 and varying values of *ϱ.*

In the last graphical example, we show quartic Julia sets in which we fixed the value of *s* and two out of three iterations’ parameters (*σ*, *ψ* and *ϱ*) and vary the remaining one. The value of the *s* parameter was set to 0.7 in each case. The generated images of the Julia sets obtained by varying *σ*, *ψ* and *ϱ* are presented in Figs 23, 24, and 25, respectively. Again, we see that each of the three iterations’ parameters impacts the size and shape of the sets. The changes are different for each of the parameters. The smallest changes are visible for varying *σ* because only for very low and very high values the set shape differs in a significant way from the sets obtained for the middle values of *σ*. On the other hand, the biggest changes are noticeable for the varying *ϱ*. The set obtained for *ϱ* = 1.0 does not remind any other set presented in this example.

## 5 Numerical results

From Sect 4, we can observe that the graphical representations of the Mandelbrot and Julia sets generated by the Picard–Thakur iteration process with *s*-convexity differ due to the varying settings of iterative parameters. To investigate the variation of size and the escaping speed, we employ two numerical measures: average escape time (AET) and non-escaping area index (NAI), as introduced in [39]. The AET provides insights into the average number of iterations performed solely for the escaping points, while NAI, ranging from [0, 1], indicates the percentage of the considered area occupied by non-escaping points (points for which the maximum of *N* iterations is performed), thereby representing the relative set size in *A*.

Because the Picard–Thakur iteration with *s*-convexity has four parameters (*σ*, *ψ*, *ϱ*, and *s*), we cannot create a single plot that shows the dependency between all the parameters and the numerical measures. Instead, we will create cross-sections using the same methodology as in [34], i.e., we will create two types of cross-sections. In the first type, we select several fixed settings of *σ*, *ψ*, *ϱ* values, and for each setting, we vary the *s* parameter with the step 0.01. Then, the data is plotted as a 2D line plot for each setting. In the second type of cross-section, we fix *s*, select several values for *ϱ*, and vary *σ*, *ψ* with the step 0.01 for each setting of *s* and *ϱ*. Then, the data is plotted as a 2D heat map, where the value of the numerical measure is mapped to colour.

The image generation algorithms were implemented in Mathematica 11.3, utilising the parallelisation feature of the Compile command, with images produced at a resolution of 800 × 800 pixels. Because the steps for the varying parameters were set to 0.01, so we created 10, 000 images for a single heat map and 100 images for a single line plot. The numerical experiments were conducted on a computer with an Intel i7-5600U processor and Windows 10 (64-bit).

### 5.1 Mandelbrot set

To generate the data for the first type of cross-section, we set *N* = 20, $A={\left[-2,2\right]}^{2}$, and the following eight settings of the fixed parameters:

- *σ* = *ψ* = *ϱ* = 0.10,
- *σ* = 0.30, *ψ* = 0.10, *ϱ* = 0.15,
- *σ* = 0.45, *ψ* = 0.30, *ϱ* = 0.25,
- *σ* = *ψ* = *ϱ* = 0.5,
- *σ* = 0.65, *ψ* = 0.45, *ϱ* = 0.35,
- *σ* = 0.85, *ψ* = 0.65, *ϱ* = 0.45,
- *σ* = 0.90, *ψ* = 0.80, *ϱ* = 0.70,
- *σ* = *ψ* = *ϱ* = 0.99.

**Fig 26:**
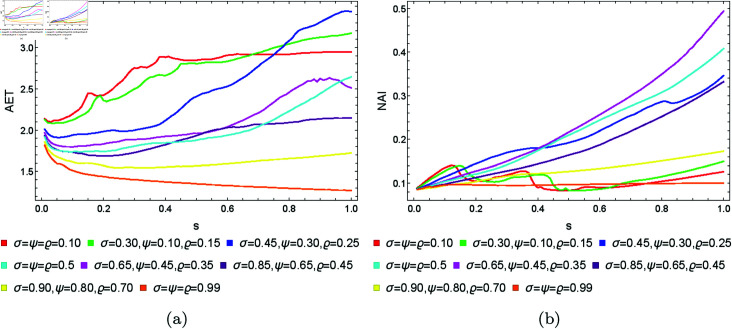
The (a) AET and (b) NAI plots for varying *s* and different fixed parameter settings for quadratic Mandelbrot sets.

**Fig 27:**
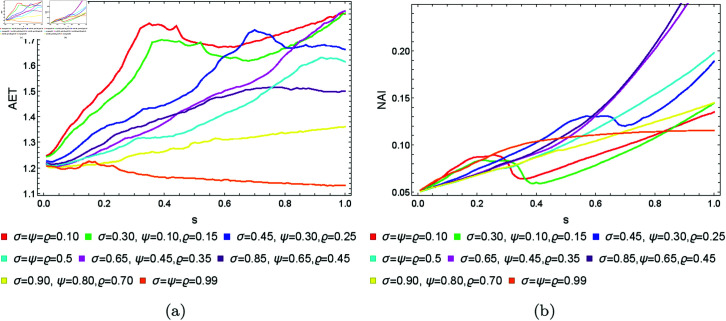
The (a) AET and (b) NAI plots for varying *s* and different fixed parameter settings for cubic Mandelbrot sets.

**Fig 28:**
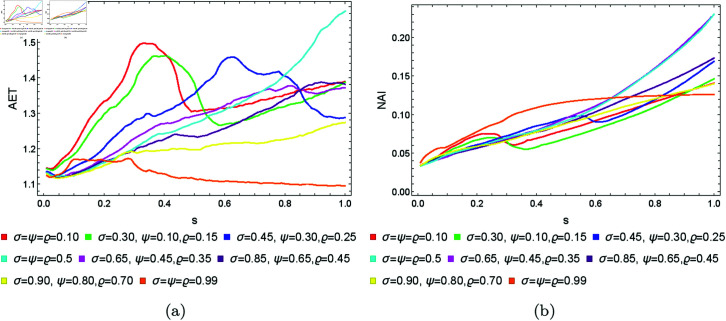
The (a) AET and (b) NAI plots for varying *s* and different fixed parameter settings for quartic Mandelbrot sets.

The obtained results for the quadratic, cubic and quartic Mandelbrot sets are presented in Figs 26, 27, and 28, respectively. We can observe that the size of the Mandelbrot sets in all three cases changes with the change in the value of parameter *s*. In case of quadratic Mandelbrot sets (Fig 26), we obtained the highest AET value (3.44) for parameter setting *σ* = 0.45, *ψ* = 0.30, *ϱ* = 0.35 and the lowest AET value (1.27) for parameter setting *σ* = *ψ* = *ϱ* = 0.99. Similarly, in the case of the NAI plot, for *σ* = 0.65, *ψ* = 0.45, *ϱ* = 0.35, we got the highest NAI value (0.49383). In the case of lower NAI values, we observe fluctuations at *σ* = *ψ* = *ϱ* = 0.10 and *σ* = 0.30, *ψ* = 0.10, *ϱ* = 0.15. In these cases, the NAI values vary between 0.08313 to 0.14022 and 0.08298 to 0.14944, respectively. In the case of cubic Mandelbrot sets (Fig 27), we observed a notably high AET value of 1.804 for the parameter setting *σ* = *ψ* = *ϱ* = 0.10, contrasted sharply with the lowest recorded AET value of 1.131 at *σ* = *ψ* = *ϱ* = 0.99. In the NAI plot, the parameter combination *σ* = 0.65, *ψ* = 0.45, *ϱ* = 0.35 yielded the highest NAI value of 0.31266. Conversely, lower NAI values exhibited significant fluctuations, particularly at *σ* = *ψ* = *ϱ* = 0.10 and *σ* = 0.30, *ψ* = 0.10, *ϱ* = 0.15, where NAI values varied from 0.05167 to 0.13489 and 0.05134 to 0.14457, respectively. For the quartic Mandelbrot sets (Fig 28), in the AET plot, we observe both irregular and gradual changes in AET values for different parameter settings. We obtained the highest AET value 1.587 for parameter setting *σ* = *ψ* = *ϱ* = 0.5, and the lowest AET value 1.172 for *σ* = *ψ* = *ϱ* = 0.99. For the NAI plot, we observe smooth behaviour except for a few parameter settings. For parameter setting *σ* = *ψ* = *ϱ* = 0.5, we obtained the highest NAI value 0.23001. In both cases, by looking at the NAI plots, we can observe that for most of the parameter settings, the size of the sets is highly dependent on the value of *s*. The higher the value of *s*, the larger the set.

**Fig 29:**
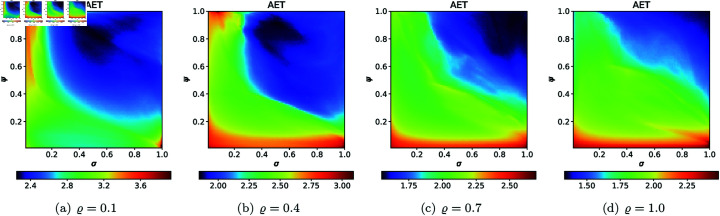
2D cross-sections of AET for quadratic Mandelbrot sets.

**Fig 30:**
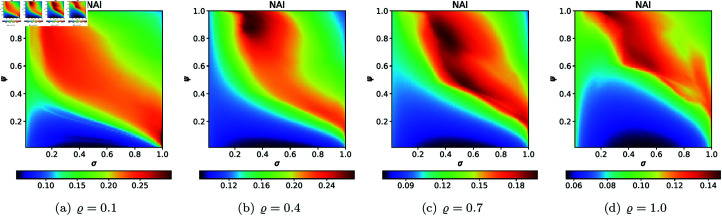
2D cross-sections of NAI for quadratic Mandelbrot sets.

For the second type of cross-section, i.e., the 2D heat maps, we set *N* = 20, $A={\left[-2,2\right]}^{2}$, *s* = 0.5, and selected the following *ϱ* values: 0.10, 0.40, 0.70, 1.0. The 2D cross-sections of AET and NAI obtained for the quadratic Mandelbrot sets are presented in Figs 29 and 30, respectively. The minimal and maximal values of AET and NAI obtained from the plots are gathered in Table 1.

**Table 1:** Minimal and maximal values of AET and NAI measures for quadratic Mandelbrot sets.

*ϱ*	min AET	max AET	min NAI	max NAI
	(*σ*, *ψ*)	(*σ*, *ψ*)	(*σ*, *ψ*)	(*σ*, *ψ*)
0.1	2.250	3.931	0.05356	0.29979
	(0.53, 0.83)	(1.0, 0.02)	(0.46, 0.01)	(1.0, 0.07)
0.4	1.848	3.091	0.08434	0.27386
	(0.53, 0.91)	(1.0, 0.01)	(0.47, 0.01)	(0.30, 0.88)
0.7	1.548	2.697	0.07132	0.19730
	(0.90, 0.98)	(1.0, 0.01)	(0.49, 0.01)	(0.42, 0.78)
1.0	1.332	2.485	0.05534	0.14728
	(1.0, 1.0)	(1.0, 0.03)	(0.53, 0.01)	(0.21, 1.0)

From plots in Figs 29 and 30, we can observe the non-linear relation between the iterative parameters and geometry of the quadratic Mandelbrot set. The AET values range between 1.332 and 3.931 when the values of *ϱ* vary from 0.1 to 1.0. We can notice that minimum AET value (1.332) obtained at *ϱ* = 1.0 (*σ* = 1.0, *ψ* = 1.0) and the maximum value of AET (3.931) obtained at *ϱ* = 0.1 (*σ* = 1.0, *ψ* = 0.02). A gradual decrease in AET values is noted as *ϱ* increases from 0.1 to 1.0, indicating that the escaping speed for the quadratic Mandelbrot set decreases with increasing values of the iterative parameter *ϱ*. Higher AET values correspond to a slower escaping speed on average. The highest NAI value 0.29979 obtained at *ϱ* = 0.1 (*σ* = 1.0, *ψ* = 0.07) indicates that the quadratic Mandelbrot set covers 29*%* of the given area. We obtained the lowest NAI value 0.05356 at *ϱ* = 0.1 (*σ* = 0.46, *ψ* = 0.01). We can observe that NAI values decrease as values of *ϱ* vary from 0.1 to 1.0.

**Fig 31:**
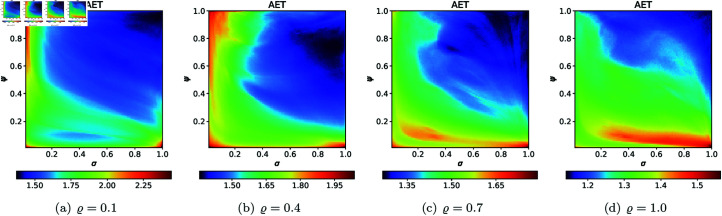
2D cross-sections of AET for cubic Mandelbrot sets.

**Fig 32:**
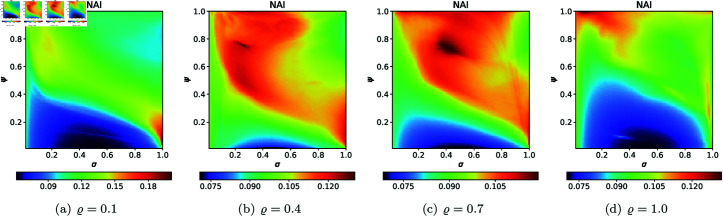
2D cross-sections of NAI for cubic Mandelbrot sets.

For the cubic Mandelbrot sets, we used the same values of *N*, *A*, *s*, and *ϱ* as we used for quadratic Mandelbrot set, to generate 2D cross-sections for AET and NAI measures. The 2D cross-sectional view of AET and NAI for the cubic Mandelbrot sets are illustrated in Figs 31 and 32, respectively. The minimal and maximal values of AET and NAI obtained from the plots are gathered in Table 2.

The plots in Figs 31 and 32 reveal a complex, non-linear relationship between the iterative parameters and the geometry of the cubic Mandelbrot set. The AET values range between 1.140 and 2.442 as *ϱ* is adjusted from 0.1 to 1.0, reflecting the set’s sensitivity to parameter changes. Specifically, the minimum AET of 1.140 is obtained at *ϱ* = 1.0 (*σ* = 1.0, *ψ* = 1.0), whereas the maximum AET of 2.442 is seen at *ϱ* = 0.1 (*σ* = 1.0, *ψ* = 0.01). This trend suggests that as *ϱ* increases, the escape rate of points within the fractal boundary decreases. In fractal dynamics, this means higher AET values correspond to regions where points are more resistant to diverging. Furthermore, examining the NAI provides insight into the spatial extent of the fractal. The maximum NAI value, 0.20097, at *ϱ* = 0.1 (*σ* = 0.01, *ψ* = 1.0), indicates that 20*%* of the given area is encompassed by the cubic Mandelbrot set. Conversely, the minimum NAI of 0.06144 at *ϱ* = 0.1 (*σ* = 0.54, *ψ* = 0.02) reflects a more compact fractal structure. This trend of decreasing NAI values as *ϱ* varies from 0.1 to 1.0 points to a progressive reduction in the fractal’s spatial spread, suggesting that higher values of *ϱ* promote a less extensive fractal formation.

**Table 2:** Minimal and maximal values of AET and NAI measures for cubic Mandelbrot sets.

*ϱ*	min AET	max AET	min NAI	max NAI
	(*σ*, *ψ*)	(*σ*, *ψ*)	(*σ*, *ψ*)	(*σ*, *ψ*)
0.1	1.375	2.442	0.06144	0.20097
	(0.99, 0.96)	(1.0, 0.01)	(0.54, 0.02)	(0.01, 1.0)
0.4	1.358	2.037	0.07089	0.13020
	(0.95, 0.75)	(1.0, 0.01)	(0.45, 0.01)	(0.26, 0.75)
0.7	1.268	1.790	0.06846	0.11903
	(1.0, 0.53)	(1.0, 0.01)	(0.51, 0.01)	(0.41, 0.74)
1.0	1.140	1.564	0.07009	0.13176
	(1.0, 1.0)	(1.0, 0.01)	(0.53, 0.02)	(0.05, 1.0)

**Fig 33:**
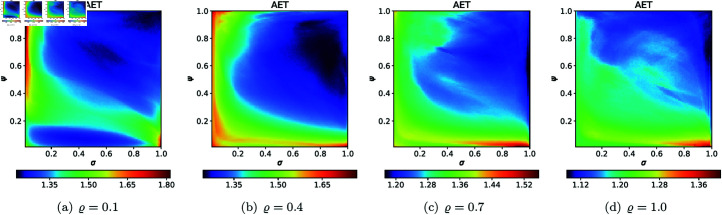
2D cross-sections of AET for quartic Mandelbrot sets.

**Fig 34:**
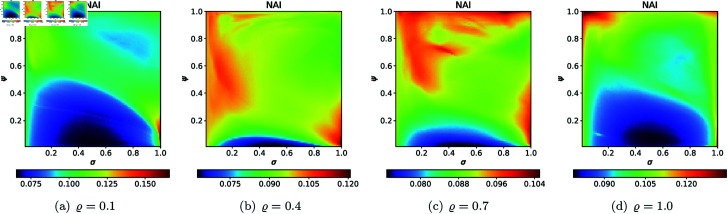
2D cross-sections of NAI for quartic Mandelbrot sets.

For the quartic Mandelbrot sets, we used the same values of *N*, *A*, *s*, and *ϱ* as we used for quadratic Mandelbrot, to generate 2D cross-sections for AET and NAI measures. The obtained 2D cross-sections of AET and NAI are shown in Fig 33 and 34, respectively. The minimal and maximal values of AET and NAI for quartic Mandelbrot sets are gathered in Table 3.

**Table 3:** Minimal and maximal values of AET and NAI measures for quartic Mandelbrot sets.

*ϱ*	min AET	max AET	min NAI	max NAI
	(*σ*, *ψ*)	(*σ*, *ψ*)	(*σ*, *ψ*)	(*σ*, *ψ*)
0.1	1.222	1.814	0.06544	0.16582
	(1.0, 1.0)	(1.0, 0.01)	(0.49, 0.04)	(0.01, 1.0)
0.4	1.245	1.767	0.06245	0.12086
	(0.85, 0.63)	(1.0, 0.01)	(0.51, 0.01)	(1.0, 0.3)
0.7	1.173	1.556	0.07292	0.10508
	(1.0, 0.56)	(0.92, 0.01)	(0.51, 0.01)	(1.0, 0.10)
1.0	1.092	1.404	0.07867	0.13459
	(1.0, 0.64)	(0.99, 0.01)	(0.52, 0.7)	(0.03, 1.0)

There is a noticeable non-linear correlation between the iterative parameters and the size of the quartic Mandelbrot set. For the NAI values, the minimum NAI value (0.06245) is obtained at *ϱ* = 0.4 (*σ* = 0.51, *ψ* = 0.01 ), and the maximum value (0.16582) at *ϱ* = 0.1 (*σ* = 0.01, *ψ* = 1.0 ). The value (0.16582) indicates that a larger size of the quartic Mandelbrot set is achieved, with 16*%* of the area covered by the points at *ϱ* = 0.1. The minimum AET value (1.092) is obtained at *ϱ* = 1.0 (*σ* = 1.0, *ψ* = 0.64) and the maximum value of AET (1.814) is obtained at *ϱ* = 0.1 (*σ* = 1.0, *ψ* = 0.01). There is a gradual change in the values of AET with the increase in the values of *ϱ*.

Overall, the interaction between the parameters *ϱ*, *σ*, and *ψ* has a significant impact on both the escape dynamics and spatial complexity of the Mandelbrot sets in all three cases discussed. These results demonstrate that parameter tuning can effectively tailor fractal geometries for specific behaviours, which may be beneficial in applications where controlled fractal densities and escape rates are desired.

### 5.2 Julia set

To generate the data for the first type of cross-section, we set *N* = 20, area $A={\left[-2,2\right]}^{2}$, *c* = − 0.57i (quadratic case), *c* = − 0.3890 − 0.1859i (cubic case), *c* = − 1.0 + 0.02i (quartic case), and the following eight settings of the fixed parameters:

- *σ* = *ψ* = *ϱ* = 0.20,
- *σ* = 0.45, *ψ* = 0.30, *ϱ* = 0.55,
- *σ* = *ψ* = *ϱ* = 0.5,
- *σ* = 0.55, *ψ* = 0.75, *ϱ* = 0.30,
- *σ* = 0.65, *ψ* = 0.45, *ϱ* = 0.35,
- *σ* = 0.85, *ψ* = 0.65, *ϱ* = 0.45,
- *σ* = 0.90, *ψ* = 0.40, *ϱ* = 0.75,
- *σ* = *ψ* = *ϱ* = 0.99.

**Fig 35:**
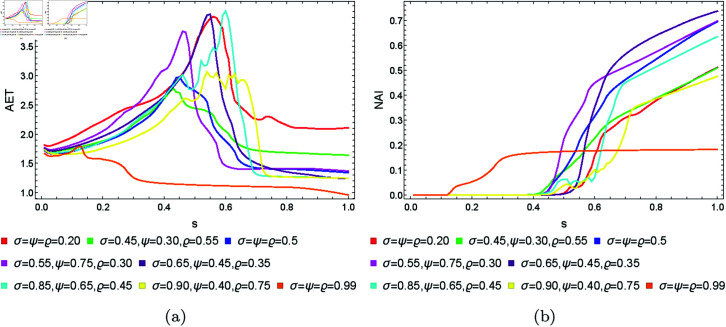
The (a) AET and (b) NAI plots for varying *s* and different fixed parameter settings for quadratic Julia sets with *c* = − 0.57i.

**Fig 36:**
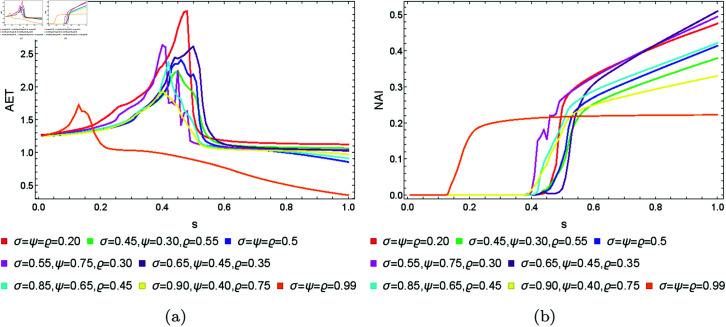
The (a) AET and (b) NAI plots for varying *s* and different fixed parameter settings for cubic Julia sets with *c* = − 0.3890 − 0.1859i.

**Fig 37:**
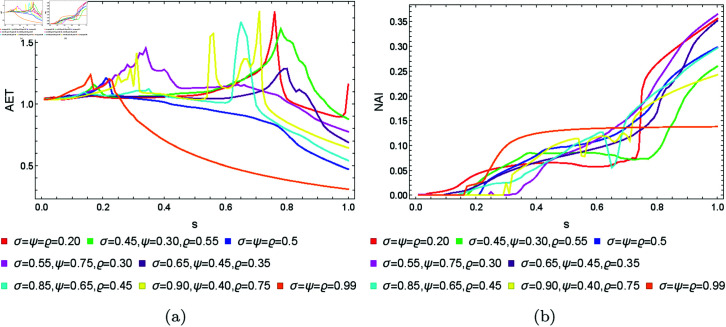
The (a) AET and (b) NAI plots for varying *s* and different fixed parameter settings for quartic Julia sets with *c* = − 1.0 + 0.02i.

The obtained plots for the quadratic, cubic and quartic Julia sets are shown in Figs 35 and 37, respectively. In the case of quadratic Julia sets, we can notice a sudden rise and then fall in the value of AET for different settings of iterative parameters, whereas NAI values change smoothly. The NAI values increase with the increase in the values of *s*. For cubic Julia sets (Fig 36), we observed significant variations in AET values for parameter *s* between 0.35 and 0.55. The maximum AET value of 3.149 occurred with the parameter settings *σ* = *ψ* = *ϱ* = 0.20, in stark contrast to the minimum recorded AET value of 0.350 at *σ* = *ψ* = *ϱ* = 0.99. In the NAI plot, the parameter combination *σ* = 0.65, *ψ* = 0.45, *ϱ* = 0.35 yielded the highest NAI value of 0.50895. For most parameter settings within the *s* range of 0 to 0.4, the NAI value remained at 0, except for *σ* = *ψ* = *ϱ* = 0.99, where a sudden increase in NAI was observed. For the quartic Julia sets, both AET and NAI plots show very random changes with the changing values of *s*. However, for all sets, the NAI values show that the higher the value of *s*, the larger the resulting set.

**Fig 38:**
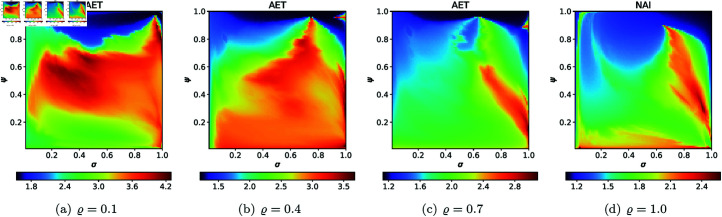
2D cross-sections of AET for quadratic Julia sets with *c* = − 0.57i.

**Fig 39:**
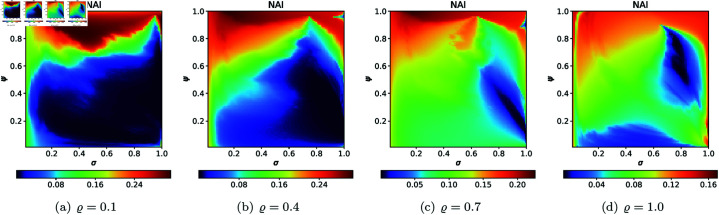
2D cross-sections of NAI for quadratic Julia sets with *c* = − 0.57i.

For the second type of cross-sections, i.e., the 2D heat maps, we set *N* = 20, $A={\left[-2,2\right]}^{2}$, *s* = 0.5, *c* = − 0.57i for the quadratic Julia set, and selected the following *ϱ* values: 0.10, 0.40, 0.70, 1.0. The 2D cross-sections of AET and NAI obtained for the quadratic Julia sets are presented in Figs 38 and 39, respectively. The minimal and maximal values of AET and NAI for the quadratic Julia sets are shown in Table 4. We can observe a non-linear behaviour between the iterative parameters and the numerical measures from the results. We can also notice that as the values of *ϱ* vary from 0.1 to 1.0, the AET values vary between 1.097 to 4.296. The minimum AET value (1.097) is obtained at *ϱ* = 1.0 (*σ* = 1.0, *ψ* = 1.0) and the maximum AET value (4.296) is obtained at *ϱ* = 0.1 (*σ* = 0.99, *ψ* = 0.82). We obtain the largest size of the Julia set at *ϱ* = 0.1, where the NAI value is 0.313198, i.e., points occupied 31*%* of the area at *σ* = 0.41, *ψ* = 0.85. The NAI value decreases as the values of *ϱ* change from 0.1 to 1.0.

**Table 4:** Minimal and maximal values of AET and NAI measures for quadratic Julia sets with *c* = − 0.57i.

*ϱ*	min AET	max AET	min NAI	max NAI
	(*σ*, *ψ*)	(*σ*, *ψ*)	(*σ*, *ψ*)	(*σ*, *ψ*)
0.1	1.538	4.296	0.00003	0.31398
	(0.98, 0.98)	(0.99, 0.82)	(1.0, 0.46)	(0.41, 0.85)
0.4	1.209	3.675	0.00005	0.30703
	(0.81, 1.0)	(1.0, 0.02)	(0.97, 0.21)	(0.15, 1.0)
0.7	1.130	3.088	0.00100	0.22594
	(0.77, 1.0)	(1.0, 0.16)	(1.0, 0.10)	(0.15, 1.0)
1.0	1.097	2.579	0.00441	0.16973
	(1.0, 1.0)	(0.04, 0.01)	(0.79, 0.57)	(1.0, 0.13)

**Fig 40:**
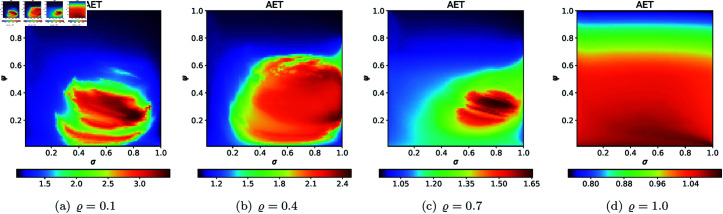
2D cross-sections of AET for cubic Julia sets with *c* = − 0.3890 − 0.1859i.

**Fig 41:**
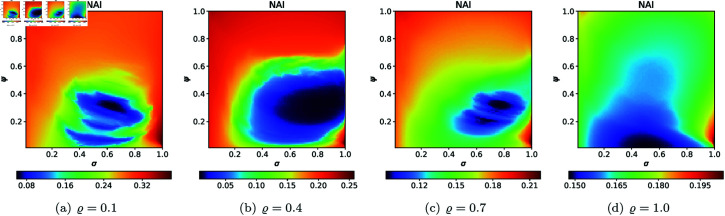
2D cross-sections of NAI for cubic Julia sets with *c* = − 0.3890 − 0.1859i.

For the cubic Julia set with *c* = − 0.3890 − 0.1859i, we employed the same values for *N*, *A*, *s*, and *ϱ* as in the quadratic case to produce 2D cross-sections of the AET and NAI measures. The resulting 2D cross-sections of AET and NAI for the cubic Julia sets are illustrated in Figs 40 and 41, respectively. The minimum and maximum values of these measures, obtained from the plots, are presented in Table 5. Based on the results, we observe that as the values of *ϱ* range from 0.1 to 1.0, the AET values vary between 0.746 and 3.475. The minimum AET value of 0.746 occurs at *ϱ* = 1.0 (with *σ* = 1.0 and *ψ* = 1.0), while the maximum AET value of 3.475 is observed at *ϱ* = 0.1 (where *σ* = 0.80 and *ψ* = 0.22). The largest Julia set size is attained at *ϱ* = 0.1, with an NAI value of 0.38051, indicating that 38*%* of the area is occupied when *σ* = 1.0 and *ψ* = 0.04. As *ϱ* increases from 0.1 to 1.0, the NAI value decreases, demonstrating a non-linear relationship between the iterative parameters and the numerical measures.

**Table 5:** Minimal and maximal values of AET and NAI measures for cubic Julia sets with *c* = − 0.3890 − 0.1859i.

*ϱ*	min AET	max AET	min NAI	max NAI
	(*σ*, *ψ*)	(*σ*, *ψ*)	(*σ*, *ψ*)	(*σ*, *ψ*)
0.1	1.052	3.475	0.05985	0.38051
	(1.0, 1.0)	(0.80, 0.22)	(0.63, 0.32)	(1.0, 0.04)
0.4	1.023	2.482	0.00829	0.25773
	(1.0, 1.0)	(1.0, 0.25)	(0.83, 0.32)	(1.0,0.03)
0.7	0.951	1.653	0.09285	0.21889
	(1.0, 1.0)	(0.80, 0.31)	(0.77, 0.31)	(1.0, 0.04)
1.0	0.746	1.114	0.14788	0.20332
	(1.0, 1.0)	(0.99, 0.01)	(0.51, 0.02)	(1.0, 0.04)

**Fig 42:**
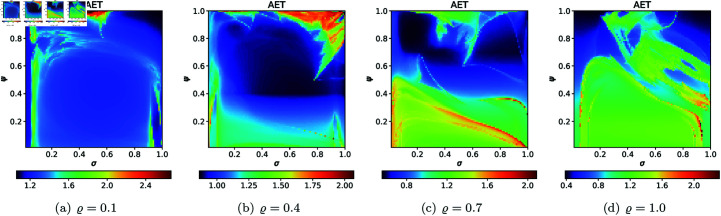
2D cross-sections of AET for quartic Julia sets with *c* = − 1.0 + 0.02i.

**Fig 43:**
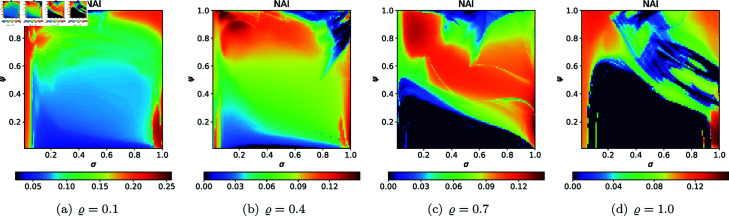
2D cross-sections of NAI for quartic Julia sets with *c* = − 1.0 + 0.02i.

For the quartic Julia set with *c* = − 1.0 + 0.02i, we used the same values of *N*, *A*, *s*, and *ϱ* as we used in the quadratic and cubic case to generate 2D cross-sections of AET and NAI measures. The 2D cross-sections of AET and NAI obtained for the quartic Julia sets are presented in Figs 42 and 43, respectively. The minimal and maximal values of the measures obtained from the plots are gathered in Table 6.

**Table 6:** Minimal and maximal values of AET and NAI measures for quartic Julia sets with *c* = −1.0 + 0.02i.

*ϱ*	min AET	max AET	min NAI	max NAI
	(*σ*, *ψ*)	(*σ*, *ψ*)	(*σ*, *ψ*)	(*σ*, *ψ*)
0.1	1.060	2.664	0.02381	0.25685
	(1.0, 1.0)	(0.48, 0.99)	(0.50, 1.0)	(0.05, 1.0)
0.4	0.862	2.072	0.00002	0.14865
	(1.0, 0.40)	(0.97, 1.0)	(0.26, 0.01)	(0.08, 0.98)
0.7	0.559	2.089	0.00002	0.14322
	(0.14, 0.86)	(1.0, 0.25)	(0.05, 0.02)	(1.0, 0.14)
1.0	0.383	2.320	0.00002	0.15260
	(0.09, 1.0)	(0.93, 0.18)	(0.05, 0.2)	(1.0, 0.07)

Similar to the quadratic Julia sets case, a non-linear behaviour between the iterative parameters and the numerical measures can be observed. The minimum AET value (0.383) was recorded at *ϱ* = 1.0 (*σ* = 0.09, *ψ* = 1.0), whereas the maximum AET value (2.664) was observed at *ϱ* = 0.1 (*σ* = 0.48, *ψ* = 0.99). The AET values decrease as *ϱ* varies from 0.1 to 1.0. From Table 6, the minimum NAI value (0.00002) can be seen at *ϱ* = 0.4, 0.7, and 1.0 for low values of *σ* and *ψ*. The maximum NAI value (0.25685) was observed at *ϱ* = 0.1 (*σ* = 0.05, *ψ* = 1.0). It can be concluded that at *ϱ* = 1.0, the points occupied the largest area, about 25*%* of the area. It is also evident that the sizes of the quartic Julia sets decrease as the *ϱ* value increases from 0.1 to 1.0.

## 6 Conclusions

In the paper, we investigated the properties of Mandelbrot and Julia sets. Our approach employed the Picard–Thakur iteration process with *s*-convexity. Using the proposed iteration process, we developed an escape criterion for generating Mandelbrot and Julia sets. Additionally, we explored how parameter variations impact the geometry of the resulting Mandelbrot and Julia set graphics. The colour, size, and shape of these sets change with adjustments in the iterative parameters, with even slight modifications leading to significant differences in their appearance. Each of the iteration’s parameters affects the set differently, and the change is different across various types of sets (quadratic, cubic, etc.). We assess these changes using the average escape time and the non-escaping area index. The results show that the dependencies between the iteration’s parameters and the numerical measures are very complex and highly non-linear.

The obtained complex fractals could further extend the capabilities of the algorithms that use Mandelbrot and Julia sets, e.g. they can expand the domain dictionary used in fractal image compression [44] or broaden the space for the initial keys used in image encryption [45]. Moreover, due to their attractive nature in the field of design [46,47], we believe that the results of this research might be beneficial for those who are interested in creating nice-looking graphics and designing printing patterns.

In the future, we plan to extend our study to include transcendental and rational-type complex functions. This could provide further insights into the broader class of fractal geometries and their applications in various scientific fields. Moreover, one can try to extend the results obtained in the paper from complex numbers to trinition numbers introduced in [48].

## Supporting information

Data 1 The raw data files used to obtain the plots in the paper. (ZIP)
